# Origin and Evolution of *Studiervirinae* Bacteriophages Infecting *Pectobacterium*: Horizontal Transfer Assists Adaptation to New Niches

**DOI:** 10.3390/microorganisms8111707

**Published:** 2020-10-31

**Authors:** Peter V. Evseev, Anna A. Lukianova, Mikhail M. Shneider, Aleksei A. Korzhenkov, Eugenia N. Bugaeva, Anastasia P. Kabanova, Kirill K. Miroshnikov, Eugene E. Kulikov, Stepan V. Toshchakov, Alexander N. Ignatov, Konstantin A. Miroshnikov

**Affiliations:** 1 Shemyakin–Ovchinnikov Institute of Bioorganic Chemistry, Russian Academy of Sciences, 117997 Moscow, Russia; petevseev@gmail.com (P.V.E.); a.al.lukianova@gmail.com (A.A.L.); mm_shn@mail.ru (M.M.S.); bygaeva.genia@gmail.com (E.N.B.); asiasay@yandex.ru (A.P.K.); 2 Department of Biology, Lomonosov Moscow State University, 119991 Moscow, Russia; 3 Federal Research Center “Kurchatov Institute”, 123182 Moscow, Russia; oscypek@yandex.ru; 4 Research Center “PhytoEngineering” Ltd., Rogachevo, 141880 Moscow Region, Russia; an.ignatov@gmail.com; 5 Winogradsky Institute of Microbiology, Federal Research Center “Fundamentals of Biotechnology”, Russian Academy of Sciences, 117312 Moscow, Russia; infon18@gmail.com (K.K.M.); eumenius@gmail.com (E.E.K.); stepan.toshchakov@gmail.com (S.V.T.)

**Keywords:** phage, *Autographiviridae*, *Pectobacterium*, tail spike, horizontal transfer, evolution, phage therapy

## Abstract

Black leg and soft rot are devastating diseases causing up to 50% loss of potential potato yield. The search for, and characterization of, bacterial viruses (bacteriophages) suitable for the control of these diseases is currently a sought-after task for agricultural microbiology. Isolated lytic *Pectobacterium* bacteriophages Q19, PP47 and PP81 possess a similar broad host range but differ in their genomic properties. The genomic features of characterized phages have been described and compared to other *Studiervirinae* bacteriophages. Thorough phylogenetic analysis has clarified the taxonomy of the phages and their positioning relative to other genera of the *Autographiviridae* family. *Pectobacterium* phage Q19 seems to represent a new genus not described previously. The genomes of the phages are generally similar to the genome of phage T7 of the *Teseptimavirus* genus but possess a number of specific features. Examination of the structure of the genes and proteins of the phages, including the tail spike protein, underlines the important role of horizontal gene exchange in the evolution of these phages, assisting their adaptation to *Pectobacterium* hosts. The results provide the basis for the development of bacteriophage-based biocontrol of potato soft rot as an alternative to the use of antibiotics.

## 1. Introduction

The potato (*Solanum tuberosum L.*) is one of the most essential food crops and is cultivated all over the world. Black leg and soft rot in potatoes inflict great losses in the production of this crop [1]. These diseases are caused by “soft rot *Pectobacteriaceae”* (SRP), which includes species of genera *Pectobacterium* and *Dickeya* [2,3]. The cells of SRP are mostly spread by contaminated seed material and can survive on other crops, wild and weedy plants, in irrigation water and on farm equipment [2,4]. The use of efficient antibacterial compounds in agriculture is restricted or limited, so there is a lack of effective methods to control soft rot [1]. The use of bacteriophages (phages) specific to the bacteria causing plant diseases is considered to be a promising strategy [5,6,7,8]. A number of successful experiments in the prevention and control of potato soft rot by applying *Pectobacterium* and *Dickeya* phages in vitro, in planta [9,10] and in the field [11,12] have been reported. However, besides traditional hurdles on production and regulation of phage-based preparations, the construction of SRP-directed phage cocktails has problems with basic requirements. The consensus of opinion on candidate phages is that they should be lytic, specific to the target bacteria, and have a reasonably broad range. Different phages should be included in the composition to reduce the formation of phage-resistant mutants of bacteria [13,14].

Recent studies have revealed the great diversity of SRP, reflected in the fact that there are currently over 30 species of *Pectobacterium* and *Dickeya* [15,16], most of them adapted to the conditions of all environments and climatic zones used for the production of potatoes.

The taxonomy of bacteriophages (and viruses in general) has also undergone recent revolutionary changes [17,18]. The order *Caudovirales* (dsDNA tailed viruses, the largest group of known bacteriophages) has been elevated to the *Uroviricota* phylum level. Correspondingly, existing and newly formed lower level taxa of phages have been elevated and separated (https://talk.ictvonline.org/taxonomy/). For instance, short-tailed phages resembling a model phage T7 are currently represented as the *Autographiviridae* family, which has nine subfamilies and 63 genera. Thus, morphologically indistinguishable phages with a similar architecture of the genome can be categorised as belonging to different genera or even subfamilies. The question of specificity and genetic diversity of SRP phages deserves a very careful investigation.

In this work, we present a comprehensive characterisation of three related lytic bacteriophages isolated in the Moscow region within a five-year period. The study describes the biological and genomic properties of *Pectobacterium* phages PP47, PP81, and Q19 with respect to their suitability for phage control applications.

The results of a comparative phylogeny of phages belonging to the subfamily *Studiervirinae* and infecting *Pectobacterium* spp., and a bioinformatic analysis of adsorption apparatus and the whole phage genome, suggest hypotheses for possible mechanisms of adaptation of these *Pectobacterium* phages to the host.

## 2. Materials and Methods

### 2.1. Bacterial Strains and Growth Conditions

Characterised propagation strains *Pectobacterium brasiliense* F157 (PB38), NCBI accession number NZ_PJDL00000000.1 and other strains and field isolates listed in Table S1 were grown at 28 °C in LB broth or LB agar plates (1.5% agar) for 24–48 h. The strains were kept in 20% glycerol at −80 °C for long-term storage.

### 2.2. Bacteriophage Isolation and Purification

Bacteriophages were isolated from samples collected in the Moscow region. The sources were washing water in a potato warehouse (geographical coordinates 56°25′28” N, 37°9′15” E) in 2014 for PP47, a sample of rotten potatoes from a dump (56°25′33” N, 37°34′13” E) in 2015 for PP81 and urban wastewater (55°40′27” N, 37°57′49” E) in 2018 for Q19. The presence of the phages in the sample was analysed using a soft agar overlay protocol [19]. Phages were propagated using *P. brasiliense* strain F157. Cell cultures grown at 28 °C to OD_600_ ~0.5 were infected with corresponding bacteriophages at a multiplicity of infection (MOI) of 0.01 and incubated for a further 4 h with moderate agitation. Cell debris were removed by centrifugation at 10,000× *g* for 20 min at 4 °C. The supernatant was passed through a 0.22 µm membrane filter. The phages were concentrated by centrifugation at 22,000× *g* for 40 min at 4 °C. The resulting pellet was resuspended in a phage buffer to a concentration ~10^9^ pfu/mL and stored at 4 °C until used.

### 2.3. Host Range of Bacteriophages

Forty strains representing different species of *Pectobacterium* and *Dickeya*, as well as soil bacteria usually accompanying soft rot infections (Table S1), were used to assess the infection range of phages.

500 µL of liquid culture of each strain was mixed with 4 mL of 0.7% soft LB agar and overlaid onto LB plates containing 1.5% agar. 20 µL of PP47, PP81 and Q19 suspensions (10^9^ pfu/mL) were spotted onto the lawns and the plates were incubated overnight at 28 °C. Bacterial susceptibility was determined by the clarification of phage application spots. Lytic ability was verified using the titration by overlay method, with the corresponding bacterial strain.

### 2.4. Biological Activity of Bacteriophages

Adsorption and one-step-growth curve tests were processed according to [9]. Bacterial strains were grown to the mid-exponential phase and infected by individual phages at MOI = 0.01. Aliquots were taken at specified intervals, diluted using a phage buffer and centrifuged at 10,000× *g* for 1 min. The titers of unadsorbed and reversibly adsorbed phages were determined by serial dilution.

The long-term effects of the phages on bacterial growth were measured by monitoring the OD_600_ for 12 h post-infection with each phage. Bacterial cells in mid-exponential phase (10^9^ cfu/mL) were mixed with the solution of phages at an MOI of 1 and diluted with LB broth. The OD_600_ of the reaction mixtures were monitored with a microplate reader (Victor, Thermo Scientific) at 28 °C over 3 h.

### 2.5. Electron Microscopy

The morphology of phages PP47, PP81 and Q19 was analysed by transmission electron microscopy (TEM). Phage suspension (~10^9^ pfu/mL) was purified by ultracentrifugation in a CsCl gradient (rotor SW28, Beckman, 22,000× *g* for 40 min at 4 °C), dialysed against the phage buffer, placed on individual copper grids, negatively stained with 1% uranyl acetate and examined using an FEI Tecnai G2 microscope at 100kV acceleration voltage. The dimensions were averaged among ~20 individually measured particles.

### 2.6. Phage Genome Sequencing and Annotation

Phage DNA was extracted using the phenol-chloroform method and fragmented with a Bioruptor sonicator (Diagenode). Paired-end libraries were constructed using a Nebnext Ultra DNA library prep kit (New England Biolabs) and sequenced on the Illumina MiSeq™ platform (Illumina) using paired 150 bp reads. After filtering using CLC Genomics Workbench 8.5 (Qiagen), overlapping paired-end library reads were merged with the SeqPrep tool (https://github.com/jstjohn/SeqPrep). Reads were assembled using CLC Genomic workbench v. 7.5. The phage genome was annotated by predicting and validating open reading frames (ORFs) using Prodigal 2.6.1 [20] and Prokka [21] pipelines. Identified ORFs were manually curated to ensure fidelity. Functions were assigned to ORFs using a BLAST search on a custom phage protein database compiled from annotated phage GenBank sequences, InterPro server (https://www.ebi.ac.uk/interpro/entry/InterPro) and HHpred server (https://toolkit.tuebingen.mpg.de) with Pfam-A_v32.0, NCBI_Consreved_Domain_v.3.16, SMART_v6.0, PRK_6.9, PDB, SCOPe70_2.07, ECOD_ECOD_F70_20190225 and COG_KOG_v1.0 databases. Custom BLAST databases were compiled using the BLAST tool (https://blast.ncbi.nlm.nih.gov/). tRNA coding regions were searched using tRNAscan-SE [22] and ARAGORN [23]. The resulting genome map was visualised in Geneious Prime, version 2020.2.3 (https://www.geneious.com). The intergenic genome regions of the phages were searched for promoters with PhagePromoter [24] in the Galaxy framework (https://galaxy.bio.di.uminho.pt/) with threshold 0.65.

### 2.7. Phylogenetic Analysis

Phage reference genomes were downloaded from NCBI GenBank (ftp://ftp.ncbi.nlm.nih.gov/genbank). Where necessary, the genomes were annotated using Prokka [21], with a custom phage protein database compiled from annotated phage GenBank sequences. A search for homologous sequences was conducted using a BLAST search and sequences found were checked for the presence of annotated homologous genes in NCBI genomes. Genes were extracted from GenBank annotations. For some unannotated sequences, ORFs were found using Glimmer [25]. ORFs were validated and corrected by comparison with known homologous genes. Protein alignments were made with MAFFT (L-INS-i algorithm, BLOSUM62 scoring matrix, 1.53 gap open penalty, 0.123 offset value) [26]. The alignments were trimmed manually and with trimAL [27] with gappyout settings. Best protein models were found with MEGAX 10.0.5 [28]. Trees were constructed using the maximum likelihood (ML) method with an RAxML program [29] and a WAG+G protein model, and the robustness of the trees was assessed by bootstrapping (1000) and with MrBayes [30,31].

### 2.8. Whole-Genome and Proteome Analysis

Average nucleotide identity (ANI) was computed using the OrthoANIu tool [32], employing USEARCH (http://www.drive5.com/usearch/) over BLAST (https://www.ezbiocloud.net/tools/orthoaniu) with default settings. Core protein extraction was performed with BPGA software [33]. Searches for homologous sequences were conducted with BLAST on custom databases based on Genbank sequences and on the nr/nt NCBI database. The VIRIDIC server (http://rhea.icbm.uni-oldenburg.de/VIRIDIC/) was employed for calculating phage intergenomic similarities (BLASTN parameters ‘-word_size 7-reward 2-penalty-3-gapopen 5-gapextend 2′) [34].

### 2.9. D Homology Modelling, Alignment and Visualisation

Protein remote homology detection, 3D structure prediction and template-based homology prediction were made using the Phyre2 protein fold recognition server (http://www.sbg.bio.ic.ac.uk/~phyre2) [35], HHpred (https://toolkit.tuebingen.mpg.de/tools/hhpred). The obtained structures were aligned and visualised with UCSF Chimera [36].

## 3. Results

### 3.1. General Properties of Pectobacterium Bacteriophages PP47, PP81 and Q19

*Pectobacterium brasiliense* (syn: *P. carotovorum* subsp. *brasiliense*) [37,38] is one of the major concerns in relation to the soft rot pathogenesis of potatoes in Central European Russia, and has been since the early 2010s [39,40]. *P. brasilense* has a certain heterogeneity in terms of its genomic and physiological properties [41,42]. Several genetically distinct strains of it were isolated in Russia [39,43], and they are used as components of enrichment culture to isolate SRP-specific bacteriophages. On the lawn of the isolation strain F157, bacteriophages formed clear plaques with a diameter of 2–3 mm for PP47 and P81, and 3-5 mm for Q19 (LB/1.5% w/v bottom agar, 0.5% w/v top agar, 28 °C). 

**Figure 1:**
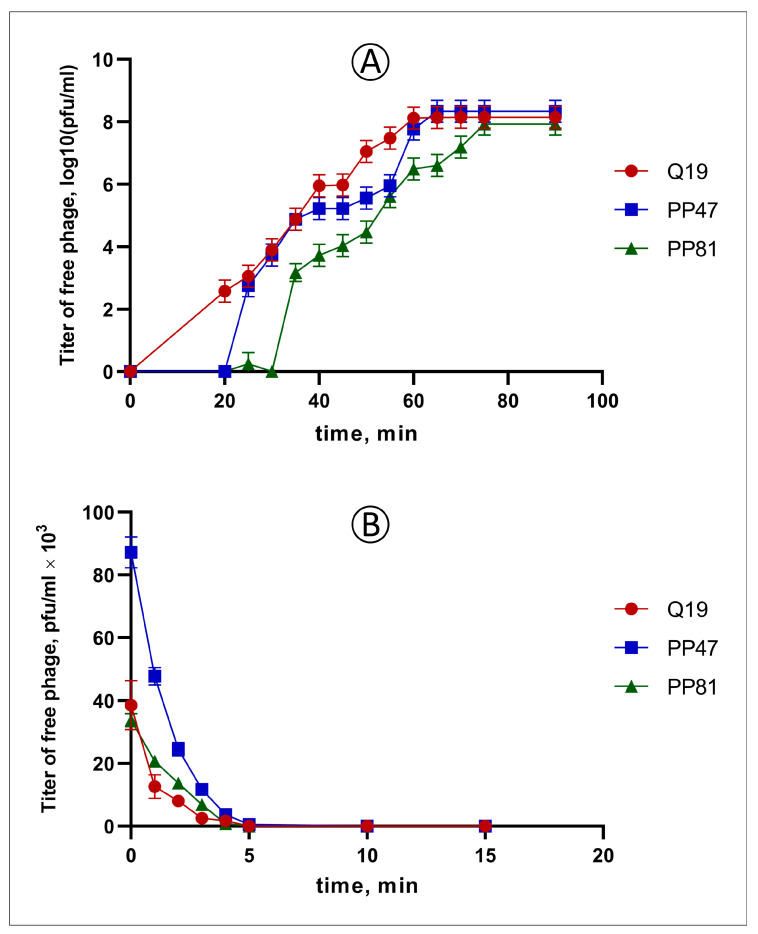
(**A**) One-step growth curve of phages Q19 (red), PP47 (blue) and PP81 (green) using *P. brasiliense* F157 as host strain in MOI = 0.01. (**B**) Adsorption of phages Q19 (red), PP47 (blue) and PP81 (green) at the surface of *P. brasiliense* F157 in MOI = 0.001.

Infectivity assays of all three phages, in standard conditions and on the same bacterial host, showed certain differences in their infection cycle. All phages demonstrated fast adsorption, in 3–5 min, and fast lysis of the host culture. Phage PP81 had a notably longer latent period and a smaller burst size (64 progeny phages/cell vs 87 for Q19 and 163 for PP47) (Figure 1).

Phages PP47, PP81 and Q19 demonstrated a fairly similar host range. All three phages infected *P. brasiliense* strains F157 and F126, while *P. brasiliense* F128 was susceptible to Q19 only, and strain F152 was resistant to all phages. All three phages infected *P. polaris* strain F109, most tested strains of *P. versatile* and some insufficiently attributed pectolytic isolates. All tested strains of *P. carotovorum, P. parmentieri* and *P. aquaticum*, and all *Dickeya* spp. used in the experiment, seemed to be resistant to PP47, PP81 and Q19 (Table S1). Therefore, in compliance with the definition of a phage suitable for phage therapy [44], the host range of the studied phages can be considered to be broad.

**Figure 2:**
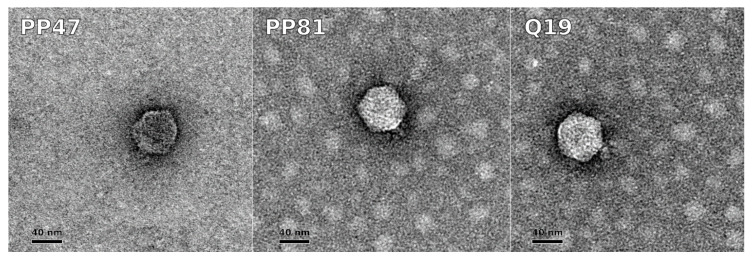
Transmission electron microscopy of bacteriophages PP47, PP81, and Q19. Staining was with 1% uranyl acetate. The scale bar is 40 nm.

The morphology of Q19, PP47 and PP81, as revealed by TEM, was typical for *Autographiviridae* phages [17,45]. The virions corresponded to Podoviral morphotype C1, with an icosahedral capsid about 60 nm in diameter and a short tail about 10 nm long. Small appendages corresponding to the phage adsorption apparatus can be seen around the tail (Figure 2).

### 3.2. Taxonomy

**Figure 3:**
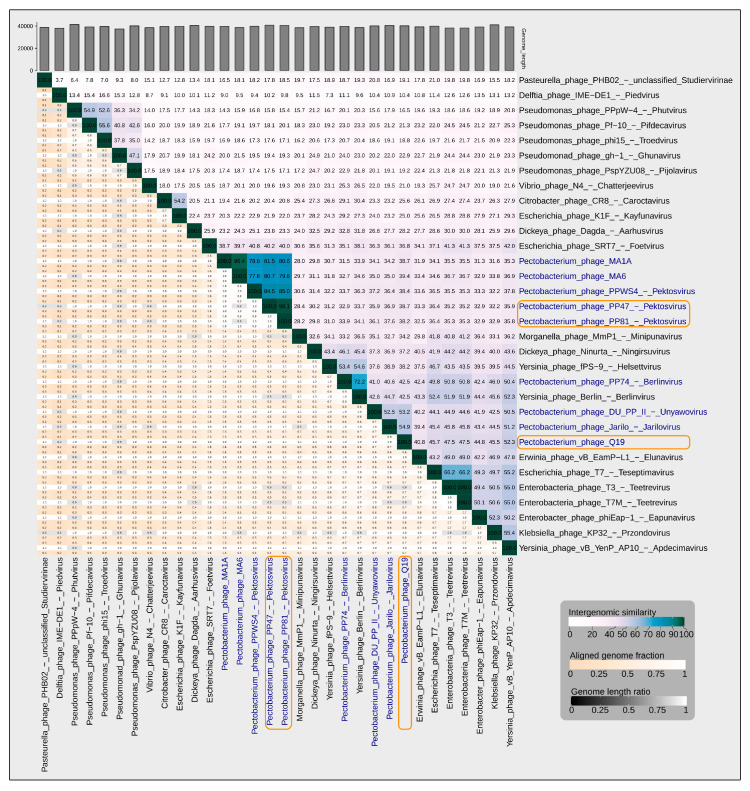
VIRIDIC generated heatmap of 30 *Studiervirinae* phages and a representative of the *Piedvirus* genus, which is closely related to *Studiervirinae*. The heatmap incorporates intergenomic similarity values (right half) and alignment indicators (left half and top annotation). In the right half, the colour coding allows a rapid visualisation of the clustering of the phage genomes based on intergenomic similarity. The numbers represent the similarity values for each genome pair, rounded to the first decimal. In the left half, three indicator values are represented for each genome pair, from top to bottom: aligned fraction genome 1 (for the genome found in this row), genome length ratio (for the two genomes in this pair) and aligned fraction genome 2 (for the genome found in this column). *Pectobacterium* phages PP47, PP81, PPWS4, MA6 and MA1A are clustered with an intergenomic similarity higher than the genus threshold of 70%. *Pectobacterium* phage Q19 has an intergenomic similarity that is lower than 70% compared to any other phage.

Intergenomic comparisons were made through calculations of average nucleotide identity using orthoANIu and whole-genome similarity by VIRIDIC, using all Genbank complete genome sequences of *Autographiviridae* phages. The latter algorithm was demonstrated to correspond to the primary classification technique used by the International Committee on Taxonomy of Viruses (ICTV), but to a higher degree [34]. The ANI calculations show a significant similarity between phages PP47 and PP81 (about 98%, Tables S2 and S3) and a lesser similarity with Q19 and all other phages (about 92%, compared to *Klebsiella* virus KP32 genome, the closest to Q19), (Table S4). These data correspond to the results of VIRIDIC analysis (Figure 3), testifying to the affiliation of PP47 and PP81 with the genus *Pektosvirus* of the *Studiervirinae* subfamily, and the affiliation of Q19 with an as yet unassigned genus. The ANI and VIRIDIC calculations point to *Pectobacterium* phages MA6 and MA1A as other members of the *Pektosvirus* genus and to the closeness of their genomes (their intergenomic similarity being higher than 95% of the species threshold). The intergenomic similarity of phages PP47 and PP81 is also higher than the species threshold. Thus, PP47 and PP81 can be considered to be strains of the same species, as well as phages MA6 and MA1A.

**Figure 4:**
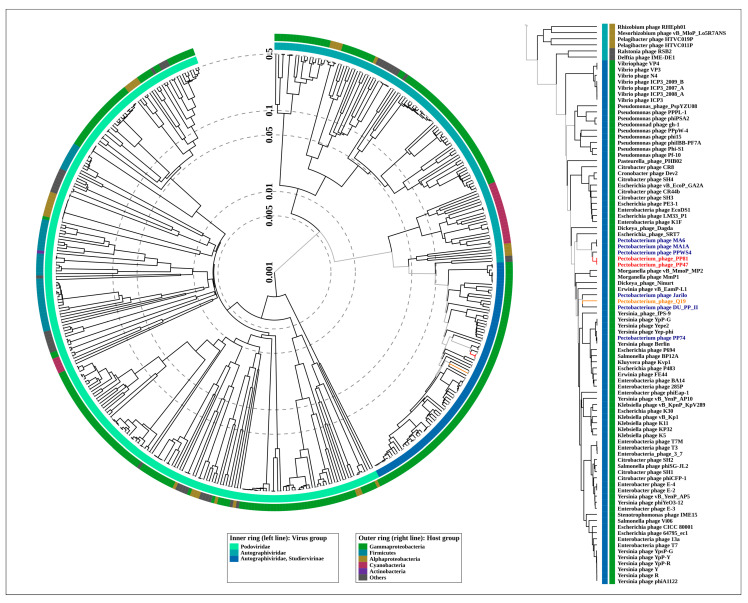
Circular proteomic tree of 447 *Podovoridae* and *Autographiviridae* phage genomes, and the *Studiervirinae* part of the tree (right) constructed using ViPTree. The branches representing *Pectobacterium* phages PP47 and PP81 are coloured red, and the branch representing *Pectobacterium* phage Q19 is coloured orange.

The proteomic tree made with ViPTree by BIONJ clustering of similar predicted protein sequences belonging to 447 phage genomes of *Podoviridae* and *Autographiviridae* families (Figure S1), and manually curated to correspond to the latest taxonomy (Figure 4), attributes the *Pectobacterium* phage Q19 to the *Studiervirinae* subfamily and also groups phages PP47, PP81, PPWS4, MA6 and MA1A together. The tree suggests the *Escherichia* phage SRT7 as a possible closest relative of *Pektosvirus* phages, and *Pectobacterium* phages Jarilo and DU_PP_II as possible closest relatives of phage Q19.

**Figure 5:**
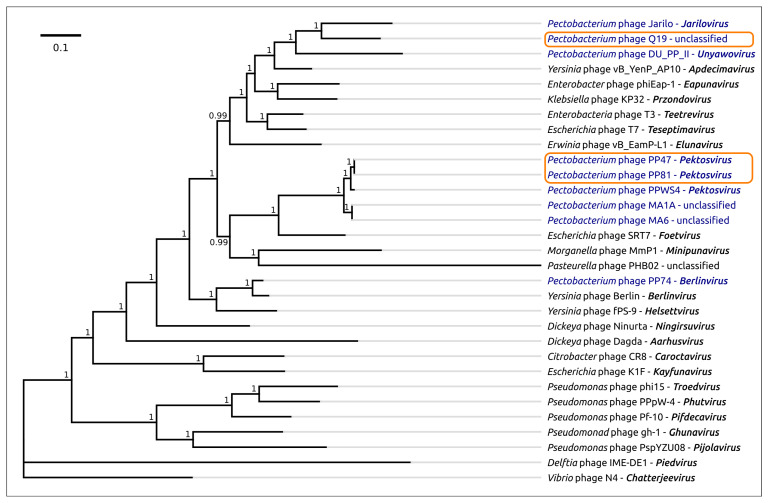
Phylogenetic tree obtained with MrBayes, based on concatenated sequences of DNA polymerase, a large subunit of terminase, a head-tail connector protein, a major capsid protein and a single-strand DNA binding protein extracted from the genomes of 29 phages, recognised by International Committee on Taxonomy of Viruses (ICTV) as master species belonging to the *Studiervirinae* subfamily, *Pectobacterium* phage PP74 of the *Berlinvirus* genus and *Delphia* phage IME-DE1. Bayesian posterior probabilities are indicated above their branch. Taxonomic classification is taken from ICTV and is shown to the right of the organism name. The scale bar shows 0.1 estimated substitutions per site and the tree was rooted to *Delphia* phage IME-DE1; of 2,000,000 generations, every 200 generations were sampled, with an average standard deviation of split frequencies of 0.0027.

The phage genomes possess a number of features which may compromise the accurate deduction of their evolutionary history [46]. These include a high level of recombination [47,48], mosaicism of the genome [49,50] and high rate of point mutations, at least for a number of proteins [51,52]. To confirm preliminary taxonomic conclusions, the phylogeny was carried out using concatenated sequences of five conserved proteins, namely DNA polymerase, a large subunit of terminase, a head-tail connector protein, a major capsid protein and a single-strand DNA binding protein. The Bayesian tree obtained for 31 phages, including 29 *Studiervirinae* phages, recognised by ICTV as master species, *Pectobacterium* phage PP74 and *Delphia* phage IME-DE1 employed as an outgroup, proposes an evolutionary history that is somewhat different to that shown in a proteomic tree (Figure 5). This tree, nevertheless, also groups phages PP47, PP81, PPWS4, M6 and MA1A as a distinct clade, and points to *Escherichia* phage SRT7 representing the genus *Foetvirus* as a sister group. In agreement with the proteomic tree and genome similarity measurements, the concatenated protein phylogeny testifies to *Pectobacteium* phages Jarilo and DU_PP_II being close relatives of phage Q19.

**Figure 6:**
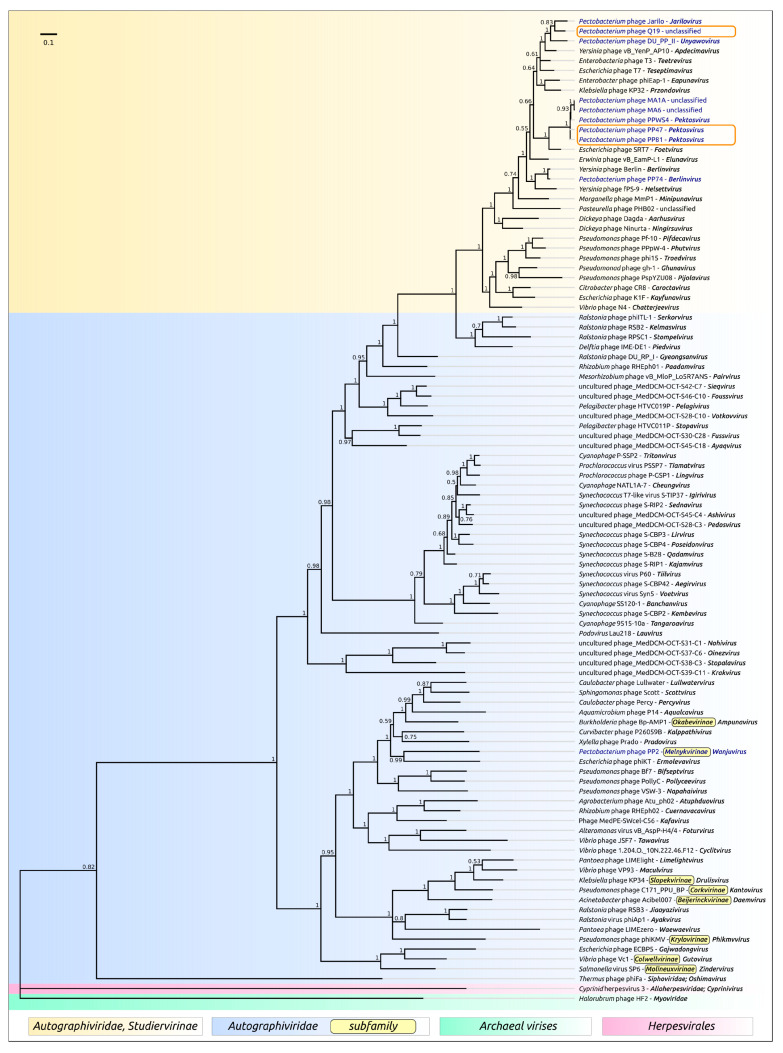
Phylogenetic tree obtained with MrBayes based on 100 terminase large subunit protein sequences. Bayesian posterior probabilities are indicated above their branch. Taxonomic classification is taken from ICTV and is shown to the right of the organism name. The scale bar shows 0.1 estimated substitutions per site and the tree was rooted to *Cyprinid* herpesvirus 3; of 2,000,000 generations, every 200 generations were sampled, with an average standard deviation of split frequencies of 0.012.

Terminase and major capsid protein (MCP) are the two most conserved proteins encoded in bacteriophage genomes, and they are often used for phage taxonomy purposes [53]. The analysis of the results of a BLAST search on the protein sequences of *Autographiviridae* indicated that terminase can be a better choice for evaluation of the phage evolutionary history than MCP at a large scale, since this protein seems to be more conservative. For the construction of the phylogeny, use was made of protein sequences extracted from 100 genomes comprising the representatives of almost all subfamilies of *Autographiviridae* and the genera not assigned to any subfamily. The tree (Figure 6) suggests early divergence of the ancestors of *Autographiviridae* into two large groups, one of which contains current *Molineuxvirinae*, *Colwellvirinae*, *Krylovirinae*, *Melnykvirinae*, *Okabevirinae* subfamilies and unassigned genera, with the other group containing the *Studiervirinae* subfamily and some unassigned genera. Interestingly, the second group also includes temperate *Pelagibacter* phages [54] and proposedly temperate cyanophages [55,56,57]. These phages can integrate their genomic DNA at tRNA sites, and the evolutionary branches of these phages are located closer to the root of the tree than those of the *Enterobacteria* phage. The topology of the *Studiervirinae* part of the terminase tree is congruent to the topology of the concatenated core proteins tree (Figure 5) and assumes the *Studiervirinae* bacteriophages infecting *Pectobacterium* have a multiple origin from different ancestral lines of phages.

### 3.3. Proteome Analysis

The proteome studies were conducted with a BLAST search with predicted protein sequences on Genbank databases. Table S5 contains the data collected from the examination of the genomes with BLAST searches on Q19, PP47 and PP81 gene products with the Genbank phage database. This data may demonstrate the complex character of evolutionary relations between these *Pectobacterium* phages. The search revealed unique proteins at the level of species and genera. In addition, the results of the BLAST examination pointed to the presence of proteins unique to several taxonomically distant *Pectobacterium* phages or proteins, which have more similarities in primary sequence with phages from comparatively taxonomically distant groups infecting *Pectobacterium*, than to taxonomically closer phages infecting other hosts. The list of proteins typical for Pectobacterial phages includes putative RNA polymerase σ54 factor (Q19 gp2) possessing a structural similarity to *E. coli* σ54 factor (HHpred probability 94%), tail spike protein, tRNA-nucleotidyltransferase and minor capsid protein. tRNA-nucleotidyltransferase and tail spike protein sequences collected the largest number of homologs from *Pectobacterium* phages of other taxa (right column in Table S5).

Interestingly, the results of a BLAST search using the nr/nt NCBI database demonstrated a greater similarity between some *Pectobacteruim* phage proteins and their bacterial homologs than that between *Pectobacteruim* phage proteins and non-*Pectobacteruim* phage proteins. This might have been a consequence of horizontal transfer. A BLAST search of the Genbank bacterial database indicated the presence of bacterial homologs for 25 of the 50 predicted proteins of Q19 and for 26 of the 55 predicted proteins of PP47. The list of these homologs contained the proteins encoded in all three phage genome regions (early, middle and late).

### 3.4. Genomic Analysis

**Figure 7:**
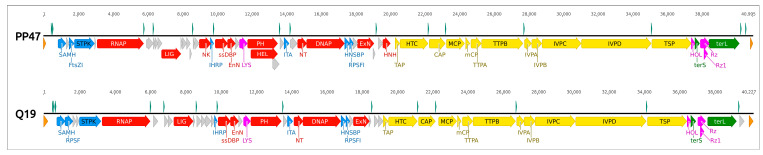
Genetic map of phages PP47 and Q19. The colors of different functional modules are as follows: yellow, morphogenesis; red, replication, transcription and nucleic acids processing; green, packaging; purple, lysis proteins; blue, regulation of host defense and metabolic processes; orange, terminal repeats; grey, hypothetical proteins. Putative transcriptional promoters, predicted with Phage Promoters, are shown above each sequence (presented as a black line) and colored cyan. Numbers above the sequences show the position in genomes. Genes’ names are as follows: SAMH, S-adenosyl-L-methionine hydrolase; RPSF, RNA polymerase σ54 factor; FtsZI, cell division FtsZ inhibitor; STPK, seryl-threonyl protein kinase; RNAP, DNA-directed RNA polymerase; LIG, DNA ligase; NK, nucleotide kinase; IHRP, inhibitor of host bacterial RNA polymerase; ssDBP, ssDNA-binding protein CDS; EnN, endonuclease I; LYS, lysozyme, N-acetylmuramoyl-L-alanine amidase; PH, DNA primase/helicase; HEL, DNA helicase; ITA, inhibitor of toxin/antitoxin system; NT, nucleotidyltransferase; DNAP, DNA polymerase; HNSBP, H-NS and tRNA binding protein CDS; RPSFI, host RNA polymerase σ70 factor inhibitor; ExN, 5′-3′ exonuclease; HNH, HNH endonuclease; TAP, tail assembly protein; HTC, head-tail connector protein; CAP, capsid assembly protein; MCP, major capsid protein; mCP, minor capsid protein; TTPA, tail tubular protein A; TTPB, tail tubular protein B; IVPA, internal virion protein A; IVPB, internal virion protein B; IVPC, internal virion protein C; IVPD, internal virion protein D; TSP, tail spike protein, SGNH hydrolase domain-containing protein; HOL, class II holin; terS, terminase small subunit; Rz, Rz lysis protein; Rz1, Rz1 lysis protein; terL, terminase large subunit.

*Pectobacterium* phages PP47 (Genbank accession KY250035), PP81 (accession KY124276) and Q19 (accession MK290739) have linear dsDNA genomes of 40,995 bp, 40,751 bp and 40,227 bp, respectively. The GC content of Q19 genome was 49.7% and the GC content of PP47 and PP81 was 48.9%. This is slightly less that the CG content (51.8–52.0%) of bacterial hosts with sequenced genomes. The genomes of PP47, PP81 and Q19 encoded 55, 54 and 50 predicted gene products (gp), respectively. The genomes were flanked with terminal repeats with a size of 151 bp (PP47, PP81) and 222 bp (Q19). The BLAST comparison of genes indicated that the genomes of PP47 and PP81 are very similar—the only difference in gene content is an extra gene*22* in PP47, encoding a hypothetical protein. The homologs of gp22 were present in related *Pectobacterium* phages MA6 and MA1A but were not found in other phages. The Q19 phage genome appears to be more distinct from PP47 and PP81. Genomic maps of the phages are shown on Figure 7.

Generally, the genome organisation of phages PP47, PP81 and Q19 is typical for *sensu lato* T7-like phages, now comprising the subfamily *Studiervirinae* within the family *Autographiviridae*. Unidirectional open reading frames can be divided into three major functional regions, relating to host conversion, DNA metabolism and particle formation. The location of predicted promoters is close for genomes of phages PP47/81 and Q19 (Figure 7) and is similar to the corresponding promoter site arrangement in the genome of the model phage T7 [58].

The structural blocks of all three phages are very similar and encode 12 proteins with predicted function and high similarity among *Studiervirinae* phages. The only exception is a putative minor capsid protein encoded downstream of a major capsid protein gene. It has no homologs in T7 but is similar to predicted minor capsid proteins in some *Studiervirinae* phages, including *Citrobacter* phage CR8 and *Klebsiella* phage KP32, belonging to genera *Caroctavirus* and *Przondovirus*, respectively.

The products of early genes are often produced immediately after infection and protect the bacteriophage DNA from bacterial defense mechanisms or adapt the host-cell metabolism to establish an efficient infection cycle [59]. These genes are most diverse within the subfamily, and the composition of the first block of genes differs for PP47/PP81 and Q19. All three phages encoded S-adenosyl-L-methionine hydrolase (first predicted gene in the genomes, gp1), homologous hypothetical proteins (gp2 in PP47/PP81 and gp3 in Q19) and serine/threonine kinase (gp4 in PP47/PP81 and gp5 in Q19). The difference in the composition of the products of early genes is the presence of a predicted cell division inhibitor FtsZ (gp3 in PP47/PP81) [60] which has no obvious homologs in the Q19 predicted proteome. Conversely, the genome of Q19 (but not PP47/PP81) encodes a putative RNA polymerase σ factor that has structural homology with bacterial σ54 enhancer-dependent σ54 transcription factor [27], revealed by HMM-HMM search with HHpred (PDB entry: 5ui5; Probability: 94.35%; E-value: 0.67; Score: 37.59). One other hypothetical protein, gp4, encoded in the early gene block of Q19, also has no phage homologs or similar structural proteins.

The middle (nucleic acid metabolism) genome regions of PP47, PP81 and Q19 are closely related. The hallmark of the *Studiervirinae* subfamily, a single subunit, DNA-dependent RNA-polymerase located in the left-most part of this region, is very conservative in all three phages. The gene for nucleotide kinase (PP47/PP81 gp18) homologous to the T7 gene 1.7 product is an enzyme distant from all other nucleotide kinases and is able to phosphorylate both dTMP and dGMP independent of divalent cations [61]. This gene was missing in Q19, as was a putative HNH endonuclease (PP47 gp38 and PP81 gp37) located at the end of the middle genome region.

Several small hypothetical proteins were present in PP47/81 and missing in Q19, or vice versa. Putative CCA-nucleotidyltransferases (tRNA nucleotidyltransferases) of PP47 (gp28) and PP81 (gp29) share 99% of amino acid identity, while the Q19 gp24 with the same function has only 48% amino acid identity with CCA-nucleotidyltransferases of PP47/81. These gene products have no direct analogues in type phage T7 and are encoded in a few *Studiervirinae* phages. However, many *Autographiviridae* phages infecting *Pectobacterium*, even attributed to different subfamilies and genera, do have CCA-nucleotidyltransferases in their predicted proteome. This feature is discussed below, as a possible hallmark of Pectobacterial *Autographiviridae*.

**Figure 8:**
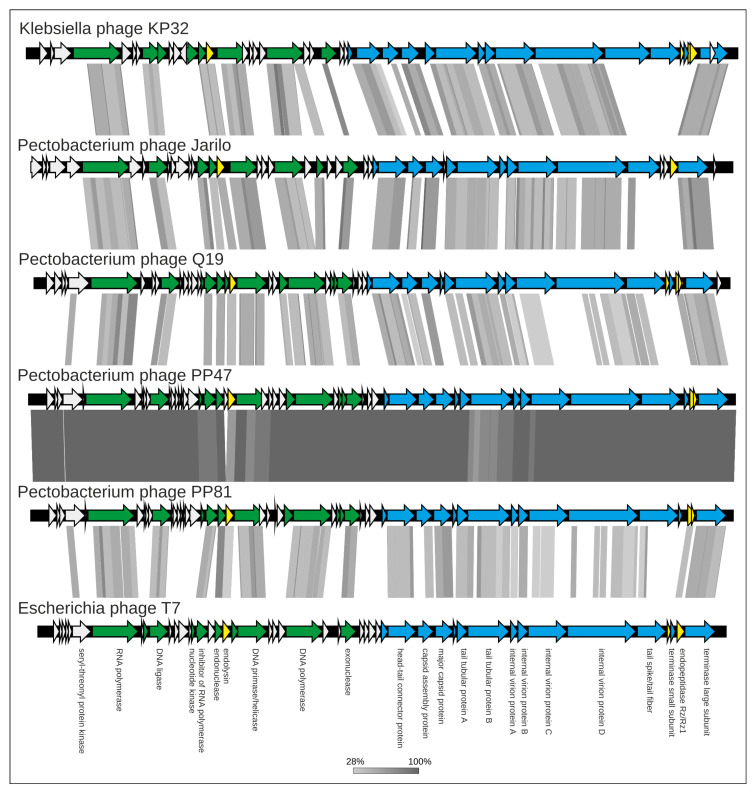
Genome sequence comparison among six *Studiervirinae* viral genomes exhibiting co-linearity detected by TBLASTX. The percentage of sequence similarity is indicated by the intensity of the grey color. Vertical blocks between analyzed sequences indicate regions with at least 28% similarity. Nucleic acid-processing genes are colored green, morphogenesis and packaging genes are colored blue and lysis genes are colored yellow. The most significant differences are observed for tail proteins and a number of hypothetical proteins of early and middle regions.

The genome comparison map made with TBLASTX comprising *Pectobacterium* phages Q19, PP47 and PP81, and phages Jarilo, *Klebsiella* phage KP32 and *Escherichia* phage T7 as representatives of different genera of *Studiervirinae*, confirms the similarity of the genomes in the regions encoding replication, structural packaging and lysis blocks (Figure 8). Non-homologous parts of the genomes are located mainly in the early gene block, hypothetical proteins of the middle region and tail spike/tail fiber proteins.

### 3.5. Tail Spike Proteins

Bacteriophage tail spike and tail fibre proteins play an important role in the phage, serving as receptor-binding proteins (RBP). Besides the function of receptor recognition, they can participate in binding and degrading lipopolysaccharides or polysaccharide capsules [51,62,63]. Some tail spikes are known to depolymerize surface polysaccharides of the host, while others show no enzymatic activity and others can deacetylate surface polysaccharides leaving the backbone of the polysaccharide intact [43,64]. The composition and structure of these RBPs are related to the host spectrum [51,65].

The bacteriophage *E. coli* T7 tail fibre is a protrusion which is about 16 nm long and 2 nm in diameter, consisting of a homo-trimer of the viral protein gp17. This protein is responsible for initial reversible host cell recognition. The following irreversible interaction with the bacterial membrane is probably mediated by one or more of the tail-tube proteins [66,67]. The tail fibers of phage T7 possess a modular structure and share a conserved N-terminal domain of ∼140 residues that anchor the tail fibre to the phage particle. Tail fibre proteins of T7-like phages are examples of a horizontal transfer of the C-terminal receptor-binding (RBP) domain [68]. The tail spike protein of *Enterobacteria* phage K1F, belonging to the *Autographiviridae* family, also possesses a modular structure with a C-terminal chaperone protein mediating homodimerization and proper folding of the catalytic endo-N trimer [69].

**Figure 9:**
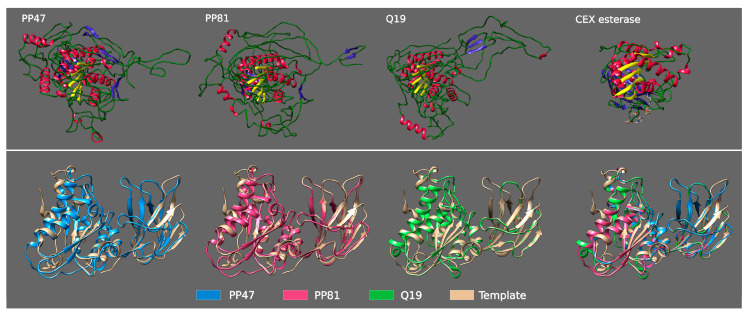
Upper picture: 3D homology modelling of tail spike proteins of *Pectobacterium* phages Q19, PP47 and PP81, and PDB structure of SGNH domain of bacterial esterase (CEX) active on acetylated mannans PDB structure 6hfz. Lower picture: 3D-alignment of modelled Q19, PP47 and PP81 tail spike SGNH domains, with SGNH domain of bacterial esterase (CEX) as a template. In the upper picture, the five parallel β-sheets intrinsic for all SGNH are colored yellow; the other β-sheets are colored blue, α-helices are colored red and coils are colored green.

Tail spikes of phages PP47, PP81 and Q19, as predicted by HMM-HMM and a BLAST homologs search, contain two identifiable parts. The N-terminal part (residues 1-159 in Q19) is structurally similar to the T7 fibre. The central part (residues 163-589 in Q19) structurally resembles the SGNH hydrolase domain and supposedly possesses deacetylation activity. This SGNH hydrolase domain is structurally similar to the gp63.1 tail spike protein of N4-like phage *Escherichia* phage vB_EcoP_G7C (HHpred Probability: 99.85%; E-value: 5.7 × 10^−18^; Score: 210.69), which is responsible for host cell recognition and attachment. G7C gp63.1 deacetylates the O-antigen of *E. coli* 4s lipopolysaccharide [64]. G7C gp63.1 is attached to the phage tail via gp66, which also participates in host cell binding. The homology modelling and structure comparison identifies the presence of the SGNH domain and the structural similarity of the central domains of these three phages’ tail spikes. The modelled structures demonstrate the presence of the SGNH hydrolase hallmark of a three-layer alpha/beta/alpha structure, where the β-sheets are composed of five parallel strands and contain catalytic residues Ser, Gly, Asn and His, which are conservative for SGNH hydrolases (shown in Figure S2) [70,71,72]. Homology recognition server Phyre2 (http://www.sbg.bio.ic.ac.uk/phyre2/) pointed to the structure of SGNH esterase (CEX) from a commensal gut bacterium as being the closest known structure (PBD structure 6hfz, confidence 100% for more than 70% of residues of C-domain) (Figure 9). A biological understanding of the removal of acetyl groups from β-mannan by esterase (CEX) is a key step toward efficient utilisation of this glycan [73].

The BLAST examination using the Genbank phage database indicated closer similarities of TSP from PP47, PP81, MA6, MA1A and PPWS4 to each other, than to other *Studievirinae* phages of *Pectobacterium* hosts. Moreover, the central part of the primary sequences of PP47, PP81 and Q19 tail spikes resemble proteins from *Pectobacterium* phages that are comparatively distant from PP47/PP81 and Q19, and that belong to *Corkvirinae*, *Melnykvirinae* and *Molineuxvirinae*. Interestingly, the tail spikes/fibre protein sequences from some phages, which were closer to *Pektosvirus* and Q19 in evolutionary terms, such as *Pectobacterium* phages Jarilo and DU_PP_II, demonstrated less similarity than those *Pectobacterium* phages belonging to *Corkvirinae*, *Melnykvirinae* and *Molineuxvirinae*. The homology modelling, the comparison of primary and secondary structure, indicated a discontinuous variation in tail spike/fibre proteins in the evolution of *Pectobacterium Autographiviridae* phages (Figure S3).

**Figure 10:**
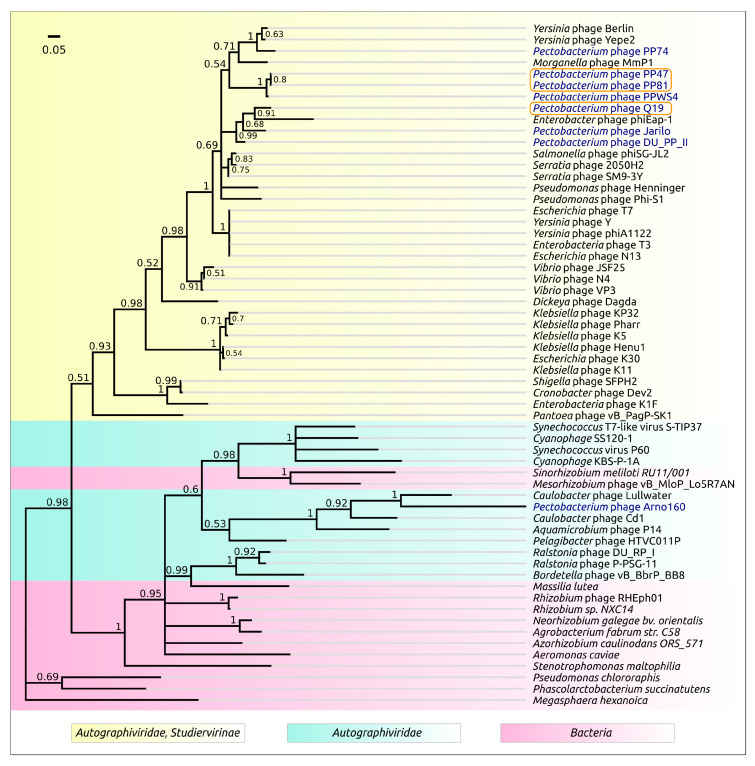
Phylogenetic tree obtained with MrBayes, based on amino acid sequences of the N-domain of tail fibre/tail spike proteins and homologous sequences obtained by a BLAST search of Genbank phage and bacterial databases. Bayesian posterior probabilities are indicated above their branch. The scale bar shows 0.05 estimated substitutions per site and the tree was rooted to *Megasphaera hexanoica*; of 2,000,000 generations, 200 generations were sampled, with an average standard deviation of split frequencies of 0.011.

**Figure 11:**
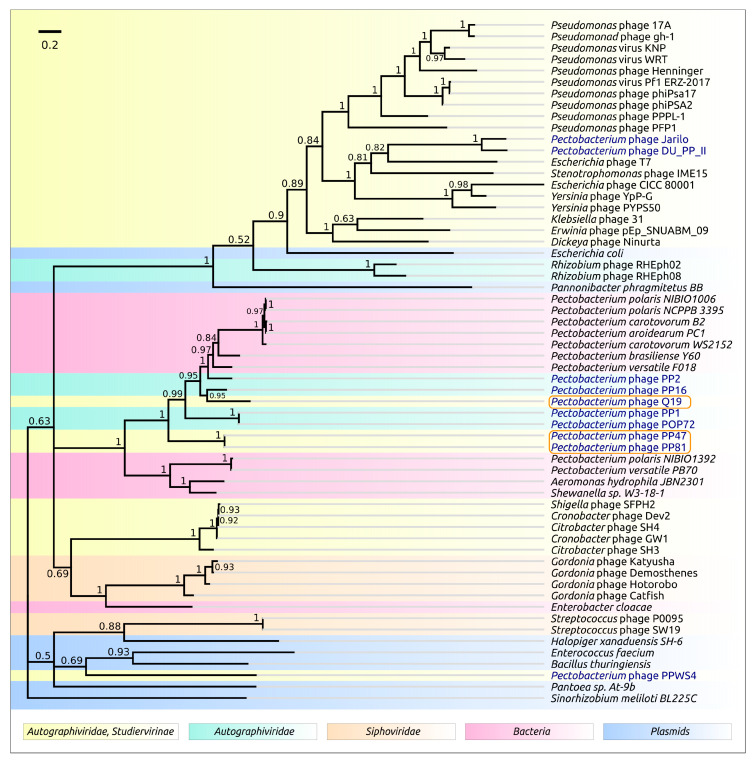
Phylogenetic tree obtained with MrBayes, based on amino acid sequences of the central domain of tail fibre/tail spike proteins and homologous sequences obtained by a BLAST search of Genbank phage and bacterial databases. Bayesian posterior probabilities are indicated above their branch. The scale bar shows 0.2 estimated substitutions per site and the tree was rooted to *Sinorhizobium meliloti BL225C* plasmid; of 2,000,000 generations, 200 generations were sampled, with an average standard deviation of split frequencies of 0.0062.

To understand the nature of the evolution of *Studiervirinae Pectobacterium* phage tail spikes, BLAST searches of protein sequences of N-part and SGNH-like domains obtained from HHpred alignment on nr/nt and Genbank bacterial databases were conducted. The searches revealed homologous sequences in the genomes of several pathogenic *Pectobacterium* strains, including strains of *P. brasiliense* and *P. versatile*, as well as in a number of other bacteria. It has been demonstrated experimentally that *P. brasiliense* and *P. versatile* serve as natural hosts for phages PP47, PP81 and Q19. The phylogenetic trees constructed with these sequences demonstrate the different evolutionary history of the T7-like N-part domain and the G7C-like SGNH domain (Figure 10 and Figure 11), suggesting the possible role of horizontal transfer in the formation of PP47, PP81 and Q19 tail spikes. *Pectobacterium* hosts of the ancestors of the phages could participate in the transfer.

**Figure 12:**
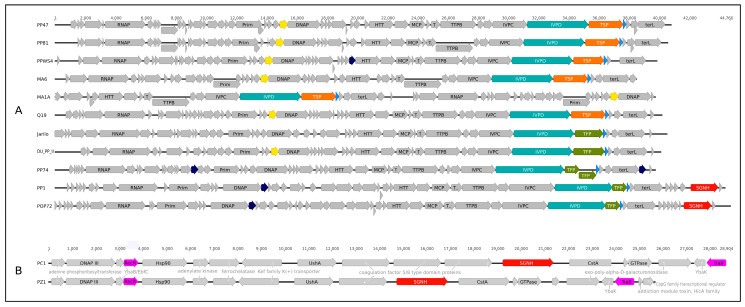
(**A**) Simplified genetic maps of *Pectobacterium Studiervirinae* phages PP47, PP81, PPWS4, MA6, MA1A, Q19, Jarilo, DU_PP_II and PP74, and phages PP1 and POP72. (**B**) Regions of *Pectobacterium aroidearum* PC1 and *Pectobacterium polaris* PZ1 containing the genes of SGNH-domain proteins, homologous to Q19 and PP47/81 tail spike proteins. The colors of different genes are as follows: orange, tail spike protein; green, tail fibre protein; cyan, tail tubular protein B; blue, holin; dark-blue, HNH endonuclease; yellow, nucleotidyltransferase; purple, recombination protein RecR and conjugal transfer protein TraB; grey, other genes. Putative functions of genes are given according to Genbank annotations and BLAST search (Tables S6–S8).

Interestingly, the location of the genes of SGNH-domain proteins, homologous to Q19 and PP47/81 tail spike proteins, in the genomes of phages of other *Autographiviridae* subfamilies is different. The genomes of *Pectobacterium* phages PP1 and POP72 of the *Molineuxvirinae* subfamily contained comparatively short 969 bp tail fibre protein genes at the end of the structural blocks and 1803 bp SGNH-domain proteins at the end of the genomes (Figure 12A). Meanwhile, the position of both tail spike protein (TSP) genes and tail fibre protein (TFP) genes is conserved in the genomes of all *Pectobacterium Studiervirinae* phages, between the genes of internal virion protein D and holin. The genome of *Pectobacterium* phage PP74 [12] includes two genes of TFP. The 100 aa-long C-end segment of the first TFP shares significant homology with the N-part of the second TFP. HMM-HMM analysis demonstrated that TFP1 resembles the first half of T7 TFP, and TFP2 the second half. Tail fibre proteins of *Pectobacterium* phages Jarilo and DU_PP_II also seem to resemble the T7 TFP in terms of structure (HHpred probability 100%). Thus, two typical structures of tail spike/fibre proteins can be distinguished for *Pectobacterium Studiervirinae* phages—T7-like tail fibre proteins for phages Jarilo, DU_PP_II and, possibly, PP74, and tail spike proteins with T7-like N-domain and SGNH-hydrolase containing C-domain for phages Q19, PP47, PP81, PPWS4, MA6 and MA1A.

The SGNH-domain proteins found in *Pectobacterium* bacteria are located within conserved regions, which also include recombination protein RecR and conjugal transfer protein TraB genes possibly involved in the recombination processes (Figure 12B).

### 3.6. tRNA-Nucleotidyltransferase

tRNA-nucleotidyltransferase (CCA-nucleotidyltransferase) is an ancient enzyme with an unusual mechanism of polymerisation, adding nucleotide triplet CCA to the 3′-end of tRNAs [74]. tRNA-nycleotidyltransferases, together with similar (by primary sequence and structurally) poly(A) polymerases, comprise a single large superfamily and can be divided into three classes, exhibiting no strong homology to one another: archaeal CCA-adding enzymes, bacterial and eukaryotic CCA-adding enzymes and bacterial poly(A) polymerases [75].

**Figure 13:**
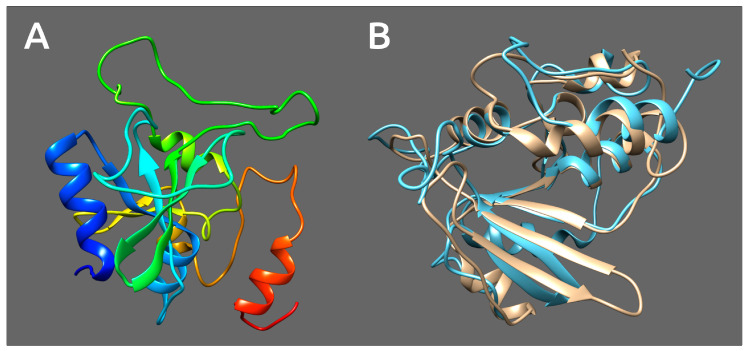
(**A**) 3D structure homology modelling of tRNA-nycleotidyltransferase from *Pectobacterium* phage PP47. The model is colored based on a rainbow gradient scheme, where the N-terminus of the polypeptide chain is colored blue and the C-terminus is colored red. (**B**) 3D-alignment of modelled tRNA-nycleotidyltransferase from *Pectobacterium* phage PP47 (colored blue) and *Pectobacerium aroidearum* PC1 (colored sand).

Analysis of annotations of Genbank genomes shows that tRNA-nucleotidyltransferase genes have been found in many bacteriophages, including 18 *Studiervirinae* phages, but, as found by BLAST search, the real number of phage genomes containing CCA-nycleotidyltransferase is higher because of a lack of annotation. Remarkably, a significant part of these phages infects *Pectobacterium* and evolutionary related plant pathogenic *Dickeya* species. It was also not possible to identify homologous genes in most of *Studiervirinae* and *Autographiviridae* genomes, nor did the analysis find proteins homologous to Q19, PP47 and PP81 putative tRNA-nycleotidyltransferases among bacteria and organisms of other kingdoms of life. However, HMM-HMM comparison demonstrated the high structural similarity of the models of these enzymes to bacterial and eukaryotic mitochondrial tRNA-nycleotidyltransferases. Interestingly, homologous modelling indicated a structural similarity between tRNA-nycleotidyltransferase from *Pectobacterium* phage PP47 and the tRNA-nycleotidyltransferase domain of a bacterial enzyme from *Pectobacerium aroidearum* PC1, in spite of the lack of any significant primary sequence similarity (Figure 13).

**Figure 14:**
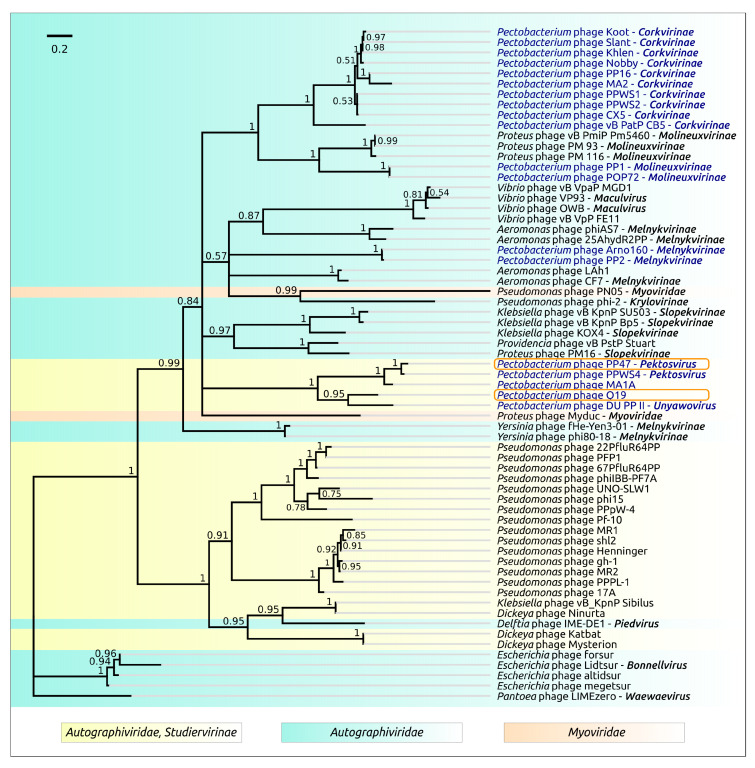
Phylogenetic tree obtained with MrBayes, based on amino acid sequences of tRNA-nucleotidyltransferase and homologous sequences obtained by a BLAST search of Genbank phage databases. Bayesian posterior probabilities are indicated above their branch. The scale bar shows 0.2 estimated substitutions per site and the tree was rooted to *Pantoea* phage LIMEzero; of 2,000,000 generations, every 200 generations were sampled, with an average standard deviation of split frequencies of 0.0071.

BLAST examination of Q19, PP47 and PP81 tRNA-nucleotidyltransferases demonstrated the sequence similarity of tRNA-nucleotidyltransferase of *Pectobacterium* phages belonging to various distant *Autographiviridae* taxa. The phylogenetic analysis performed with extracted, annotated and homologous proteins grouped the tRNA-nycleotidyltransferase sequences belonging to Q19, *Pektosvirus* and *Unyawovirus* of the *Studiervirinae* subfamily in one clade with *Corkvirinae*, *Melnykvirinae*, *Molineuxvirinae* and *Slopekvirinae*, and separately from other *Studiervirinae* sequences (Figure 14).

## 4. Discussion

### 4.1. Origin, Phylogeny and Taxonomy

The whole-genome comparisons, including VIRIDIC and ANI, as well as phylogenetic studies, indicate that *Pectobacterium* bacteriophages of the *Studiervirinae* subfamily have a complex origin, comprising several independent lines of descent. From this point of view, they share a common feature of phages, noted by Hans-Wolfgang Ackermann: “Bacteriophages are polyphyletic, arose repeatedly in different hosts” [76]. At the moment, they include the genera of *Pektosvirus* (line 1), *“Q19-virus”*, *Jarilovirus*, *Unyawovirus* (line 2) and *Pectobacterium* phage PP74 (*Berlinvirus*) (line 3). *“Q19-virus”*, *Jarilovirus* and *Unyawovirus* seem to form a monophyletic group, and *Yersinia* phage vB_YenP_AP10 of the *Apdecimavirus* genus appears to be their closest related classified phage. Phages PP47 and PP81, together with *Pectobacterim* phages PPWS4, MA6 and MA1A, form the genus of *Pektosvirus*. *Escherichia* phage SRT of the *Foetvirus* genus appears to be the closest known relative of *Pektosvirus* phages and shares with them the last common ancestor.

According to ICTV rules, phages PP47 and PP81 seem to be two strains of the same species, since their intergenomic similarity is higher than 95%. MA6 and MA1A *Pektosvirus* phages can be strains of the same species clonal group. Phage PP47 differs from PP81 in terms of proteome composition, although these phages are members of the same clonal group—the PP47 genome contains one more gene-encoding hypothetical protein that may affect the observed course of infection. Actually, PP47 and PP81 do have differences in behaviour, e.g., in infection cycle.

The origin of *Studiervirinae* is related to the early divergence of *Autographiviridae* into two large clades, one of which includes modern *Studiervirinae* phages, cyanophages, *Pelagibacter* phages and other groups, as may concluded from terminase (Figure 6) and proteome (Figure 4 and Figure S1) phylogeny.

### 4.2. Genome, Adsorption Apparatus and Horizontal Transfer

The genome organization of Q19, PP47, PP81 and other *Pectobacterium Studiervirinae* phages shares many common features with T7, T3 and other related phages, but also has specific variations, including the presence of unique genes at the level of both genera and species, the length and composition of terminal repeats and non-coding sequences, and small variations in GC-content. 

While the genome organization and protein composition of *Pectobacterium Studiervirinae* phages have much in common with their phylogenetic relatives, the phages share similarities in a number of proteins with other taxonomically distant *Pectobacterium* phages. Probably, those proteins can be important in terms of specificity to the *Pectobacterium* host. An intriguing example of tRNA-nucleotidyltransferase found in 37 of 46 Pectobacterium phages belonging to different *Autographiviridae* subfamilies, and only in 60 of more than 500 remaining *Autographiviridae* phages, raises questions about the role of this enzyme in phage infection and the possibility to use this finding for practical purposes. The presence of tRNA-nucleotidyltransferase in the bacterial genome was shown to have importance for phage reproduction [77]. tRNA-nucleotidyltransferases participate in various processes in the cell and influence bacterial growth [78,79] and interact with other bacterial proteins [80]. The biological functions, origin and evolution of tRNA-nucleotidyltransferase and other specific genes of *Pectobacterium* phages can be studied in further research.

We suggest that at least some *Pectobacterium* phage specific proteins were acquired by horizontal transfer, which contributes significantly to phage evolution [52,81,82,83,84]. Bacteriophages can mediate the processes involved with horizontal transfer in bacteria [82,83,85] and bacteriophage-mediated horizontal transfer can override the mutations [84]. It has been shown that processes such as recombinations in phage genomes [52] and point mutations also drive phage evolution [86]. It would be interesting to study the possible effects of convergent evolution on *Pectobacterium* phage genes.

The adsorption apparatus has a special meaning for host specificity [87,88,89,90,91] and the mechanism of developing such specificity to certain hosts is a matter of fundamental and applied interest. Horizontal transfer of tail fibre (tail spike) protein modules appears to be an important instrument for adaptation to new hosts [68,89]. As has been shown by the homology search, and by the structural and phylogenetic analysis, of the current research, *Pectobacterium* tail spike proteins can be formed with the assistance of horizontal transfer and the involvement of *Pectobacterium* hosts. Further research into various aspects of the evolution of adsorption apparatus can facilitate phage therapy.

## 5. Conclusions

*Pectobacterium* bacteriophages of the *Studiervirinae* subfamily have a complex origin. There are three independently evolved lines that can be distinguished. One of monophyletic group includes *Pectobacterium* phages Q19, phage Jarilo of the *Jarilovirus* genus and DU_PP_II of the *Unyawovirus* genus. Another group comprises *Pectobacterium* phages PP81, PP47, PPWS4, M6A and MA1A of the *Pektosvirus* genus and the third group is represented by *Pectobacterium* phage PP74 of the *Berlinvirus* genus. Phage Q19 represents a new genus not previously described. Phages PP47 and PP81, as well as phages M6A and MA1A, seem to represent clonal groups. Phages Q19, PP47 and PP81 infect a broad spectrum of related *Pectobacterium* hosts and possess a similar tail spike protein, which could be the consequence of exchange with other phages infecting *Pectobacterium* hosts with participation of bacterial hosts. Horizontal transfer can be the reason for the similarity of a number of genes of taxonomically distant *Pectobacterium* phages. Studying the processes of genome formation and, in particular, the adsorption apparatus can assist the search for, and design of, new phages for effective phage therapy.

## Supplementary Materials

The following are available online at https://www.mdpi.com/2076-2607/8/11/1707/s1: Figure S1: Proteomic tree of 447 *Podovoridae* and *Autographiviridae* phage genomes constructed using ViPTree. Figure S2: Alignment of secondary structures of phage tail spike proteins and SGNH-hydrolase made with UCSF Chimera, and possible active centre and conserved residues (circled with red) found by comparison with published structures. Figure S3: 3D structures of tail spike and tail fibre proteins of *Pectobacterium* phages Q19, Jarilo, DU_PP_II, PP47, PP81 and PPWS4 obtained by homology modelling and MAFFT alignment of primary sequences of tail spike and tail fibre proteins, and distance matrix for the proteins. Jarilo and DU_PP_II are suggested to be the closest relatives of Q19, and phages PP47, PP81 and PPWS4 are the members of the *Pektosvirus* genus. The models are colored based on a rainbow gradient scheme, where the N-terminus of the polypeptide chain is coloured blue and the C-terminus is colored red. Table S1: Infectious range of *Pectobacterium* phages PP47, PP81 and Q19. Table S2: Average nucleotide identity (ANI) between *Pectobacterium* phage PP47 and all *Autographiviridae* phage genomes deposited in the NCBI GenBank (threshold 0.5). Table S3: Average nucleotide identity (ANI) between and all *Autographiviridae* phage genomes deposited in the NCBI GenBank (threshold 0.5). Table S4: Average nucleotide identity (ANI) between *Pectobacterium* phage Q19 and all *Autographiviridae* phage genomes deposited in the NCBI GenBank (threshold 0.5). Table S5: The results of the BLAST examination of Q19 and PP47 gene products using the Genbank phage database (Bit-score > 40). Table S6: Functional assignments of *Pectobacterium* phage PP47 genes. Table S7: Functional assignments of *Pectobacterium* phage PP81 genes. Table S8: Functional assignments of *Pectobacterium* phage Q19 genes.
