# Supplementary material for: Origin and Evolution of Studiervirinae Bacteriophages Infecting Pectobacterium: Horizontal Transfer Assists Adaptation to New Niches
Source: Microorganisms. 2020 Oct 31;8(11):1707. doi: 10.3390/microorganisms8111707 (PMC7693777; doi:10.3390/microorganisms8111707)
Supplement: Supplementary file 1 [file microorganisms-08-01707-s001.zip › Suppl_Table_S5_Protein_BLAST_search.docx]

|  | Unique proteins for the phage | Unique proteins for the members of the genus | Unique proteins for *Pectobacterium* phages of different *Autographiviridae* taxa | Proteins more similar to *Pectobacterium* phages of distant genera than to closer non-*Pectobacteirum* phages |
| --- | --- | --- | --- | --- |
| Q19 | gp04—hypothetical protein  gp07—hypothetical protein  gp09—hypothetical protein  gp15—hypothetical protein  gp19—hypothetical protein | | gp2—RNA polymerase σ54 factor  gp11—hypothetical protein | gp24—tRNA-nucleotidyltransferase  gp26—H-NS and tRNA binding protein  gp37—minor capsid protein  gp44—tail spike protein  gp48—Rz1 lysis protein |
| PP47 | gp5—hypothetical protein  gp12—hypothetical protein  gp14—hypothetical protein | gp8—hypothetical protein  gp10—hypothetical protein  gp13—hypothetical protein  gp15—hypothetical protein  gp16—hypothetical protein  gp22—hypothetical protein  gp36—hypothetical protein |  | gp29—tRNA-nucleotidyltransferase  gp32—H-NS and tRNA binding protein  gp34—hypothetical protein  gp43—minor capsid protein  gp44—tail tubular protein A  gp51—class II holin  gp51—terminase small subunit  gp54—Rz1 lysis protein |
