# Supplementary material for: Origin and Evolution of Studiervirinae Bacteriophages Infecting Pectobacterium: Horizontal Transfer Assists Adaptation to New Niches
Source: Microorganisms. 2020 Oct 31;8(11):1707. doi: 10.3390/microorganisms8111707 (PMC7693777; doi:10.3390/microorganisms8111707)
Supplement: Supplementary file 1 [file microorganisms-08-01707-s001.zip › Suppl_Table_S1_Infect_range.docx]

|  | **Strain** | **Alternative name** | **Origin** | **Species** | **Genome** | **Q19** | **PP81** | **PP47** |
| --- | --- | --- | --- | --- | --- | --- | --- | --- |
| 1 | F002 | PB69 | Moscow Region | *P.versatile* | NZ_PDVY00000000.1 | + | + | + |
| 2 | F016 | 5 | Moscow Region | *P. versatile* |  |  |  |  |
| 3 | F018 | 211 / I5 | Moscow Region | *P. versatile* |  | + | + | + |
| 4 | F020 | 523 / I6 | Moscow Region | *P. versatile* |  | + | + | + |
| 5 | F021 | 526 / I4 | Moscow Region | *P. versatile* |  | + | + | + |
| 6 | F022 |  | Moscow Region | unidentified pectolytic isolate |  | + |  |  |
| 7 | F034 | B9 | Kaluga Region | *Pectobacterium sp.* |  |  |  |  |
| 8 | F035 | B7 | Kaluga Region | unidentified pectolytic isolate |  |  |  |  |
| 9 | F040 | 524 / H4 | Moscow Region | *P. versatile* |  | + | + | + |
| 10 | F051 | 524 / H3 | VNIIF | *Pectobacterium sp.* |  | + |  |  |
| 11 | F058 | 512 / I1 | Moscow Region | *P. carotovorum* |  |  |  |  |
| 12 | F064 | 520 / F6 | Moscow Region | *P. carotovorum* |  |  |  |  |
| 13 | F082 | H4 | Moscow Region | *Dickeya sp.* |  |  |  |  |
| 14 | F100 | 2.3 / F2 | VNIIF | *P. carotovorum* |  |  |  |  |
| 15 | F106 |  | Moscow Region | unidentified pectolytic isolate |  |  |  |  |
| 16 | F109 | 35 / E6 | Ethiopia | *P. polaris* |  | + | + | + |
| 17 | F131 | 12a | VIZR | *P. versatile* | NZ_PDVW00000000.1 | + | + | + |
| 18 | F135 | 2.1 / F5 | Moscow Region | *P. versatile* | NZ_PDVX00000000.1 | + | + | + |
| 19 | F126 | C4 | Samara Region | *P. brasiliense* | RRYQ00000000 | + | + | + |
| 20 | F127 | B5 | Kaluga Region | *D. dianticola* |  |  |  |  |
| 21 | F128 | B3 | Samara Region | *P. brasiliense* |  | + |  |  |
| 22 | F140 |  | Moscow Region | *P. carotovorum* |  |  |  |  |
| 23 | F148 | PB20 | Moscow Region | *P. parmentieri* | NZ_PDDJ00000000.1 |  |  |  |
| 24 | F149 | PB21 | Moscow Region | *P. parmentieri* |  |  |  |  |
| 25 | F152 | PB29 | Moscow Region | *P. brasiliense* | PJDM00000000 |  |  |  |
| 26 | F155 | PB36 | Moscow Region | *D. solani* |  |  |  |  |
| 27 | F157 | PB38 | Moscow Region | *P. brasiliense* | PJDL00000000 | + | + | + |
| 28 | F160 | NCPPB312 | VKPM | *P. carotovorum* | NZ_JQHJ00000000.1 |  |  |  |
| 29 | F162 | SCRI1043 | Scotland | *P. atrosepticum* | NC_004547 |  |  |  |
| 30 | F163 | 21A | Belarus | *P. atrosepticum* | NZ_CP009125 |  |  |  |
| 31 | F165 |  |  | unidentified pectolytic isolate |  |  |  |  |
| 32 | F166 |  |  | unidentified pectolytic isolate |  |  |  |  |
| 33 | F169 |  | Moscow Region | unidentified pectolytic isolate |  |  |  |  |
| 34 | F171 | 2019.19-4 | Moscow Region | *P. polaris* |  |  |  |  |
| 35 | F174 | 2019.14 | Tver Region | *P. carotovorum* |  |  |  |  |
| 36 | F175 |  | Moscow Region | unidentified pectolytic isolate |  |  |  |  |
| 37 | F177 |  | Moscow Region | unidentified pectolytic isolate |  |  |  |  |
| 38 | F182 | 2019.41 | Moscow Region | *P. polaris* |  |  |  |  |
| 39 | F189 |  | Moscow Region | unidentified pectolytic isolate |  |  |  |  |
