# Supplementary figures and images for: Origin and Evolution of Studiervirinae Bacteriophages Infecting Pectobacterium: Horizontal Transfer Assists Adaptation to New Niches
Source: Microorganisms. 2020 Oct 31;8(11):1707. doi: 10.3390/microorganisms8111707 (PMC7693777; doi:10.3390/microorganisms8111707)

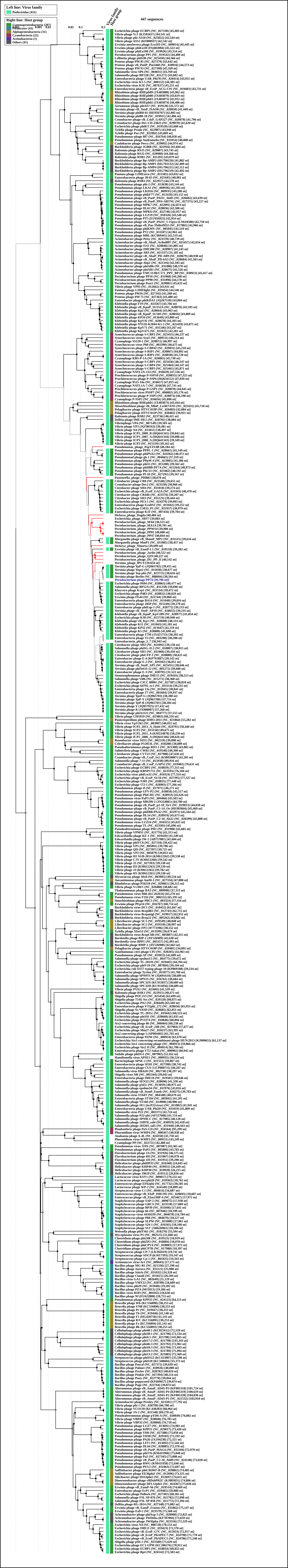

Supplement: Supplementary file 1 [file microorganisms-08-01707-s001.zip › Suppl_Fig_S1_ViP_tree.png]

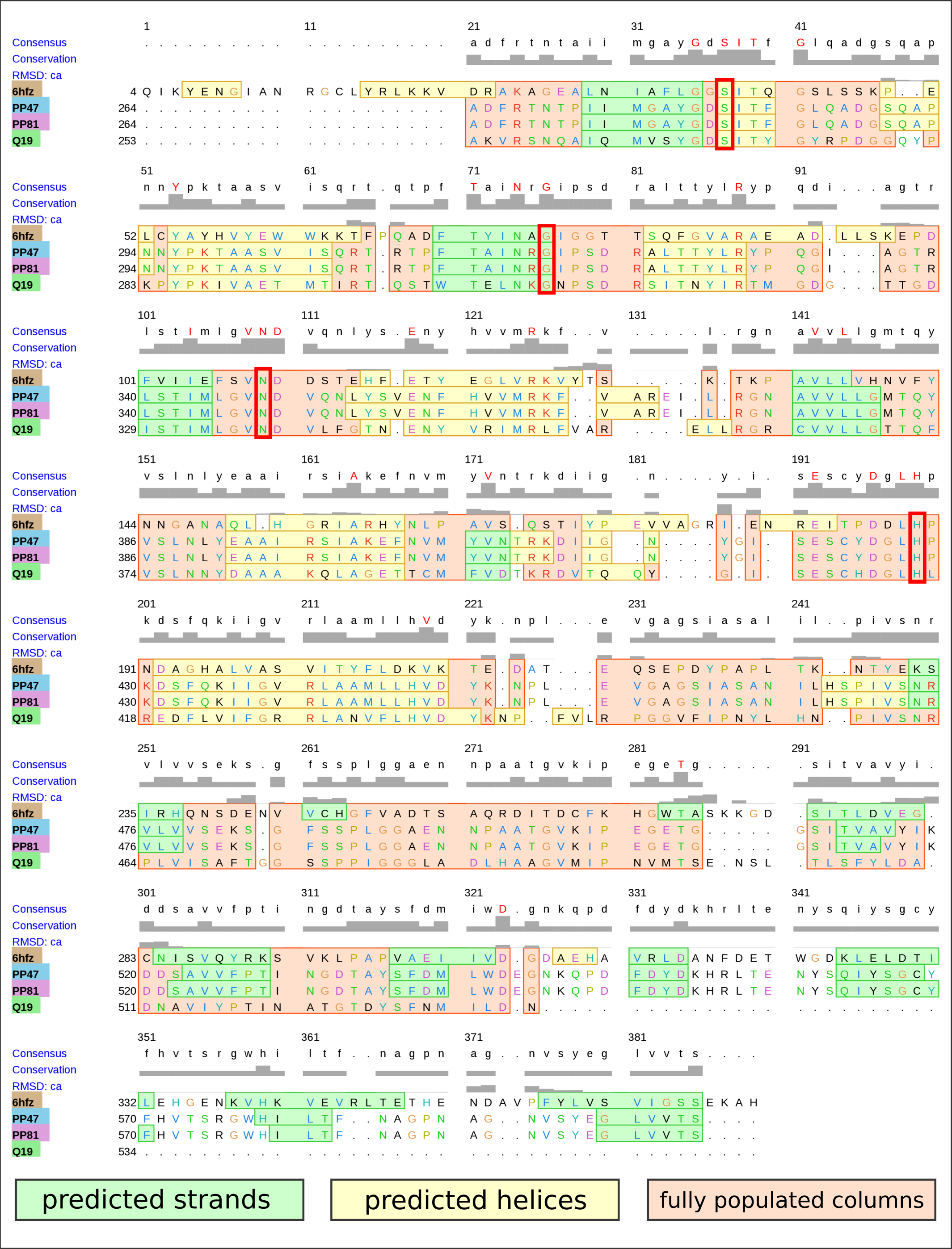

Supplement: Supplementary file 1 [file microorganisms-08-01707-s001.zip › Suppl_Fig_S2_2ry_TSP-SGNH.png]

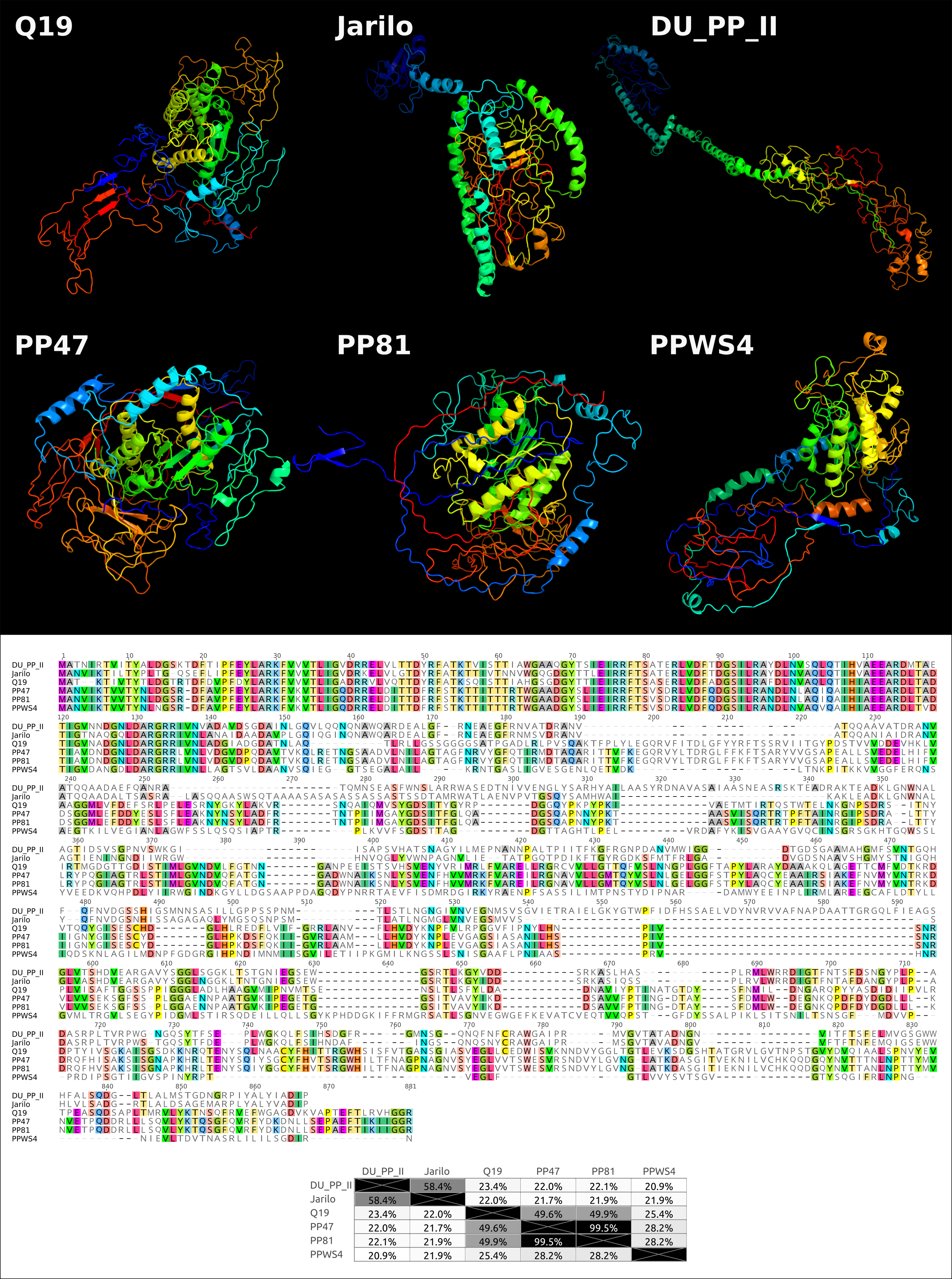

Supplement: Supplementary file 1 [file microorganisms-08-01707-s001.zip › Suppl_Fig_S3_TSP.png]
